# The impact of an educational intervention on physician leadership competencies among rural and remote primary care doctors in Aceh, Indonesia

**DOI:** 10.1108/LHS-02-2023-0011

**Published:** 2023-08-03

**Authors:** Fury Maulina, Mubasysyir Hasanbasri, Jamiu O. Busari, Fedde Scheele

**Affiliations:** Faculty of Health, Medicine and Life Sciences, School of Health Professions Education, Maastricht University, Maastricht, The Netherlands and Department of Public Health, Faculty of Medicine, Universitas Malikussaleh, Lhokseumawe, Indonesia; Department of Biostatistics, Epidemiology and Population Health, Faculty of Medicine, Public Health and Nursing, Gadjah Mada University, Yogyakarta, Indonesia; Department of Educational Development and Research, Maastricht University, Maastricht, The Netherlands and Department of Pediatrics, Dr Horacio E Oduber Hospital, Oranjestad, Aruba; Athena Institute for Transdisciplinary Research, Vrije Universiteit Amsterdam, Amsterdam, The Netherlands and Research in Education, Amsterdam UMC Locatie VUmc, Amsterdam, The Netherlands

**Keywords:** Leadership, Education, Doctors, Primary care, Rural areas

## Abstract

**Purpose:**

This study aims to examine how an educational intervention, using the lens of the LEADS framework, can influence the development of primary care doctors’ leadership skills in Aceh, Indonesia. In order to persevere in the face of inadequate resources and infrastructure, particularly in rural and remote settings of low- and middle‐income countries, physicians require strong leadership skills. However, there is a lack of information on leadership development in these settings.

**Design/methodology/approach:**

This study applied an educational intervention consisting of a two-day workshop. The authors evaluated the impact of the workshop on participants’ knowledge and skill by combining quantitative pre- and post-intervention questionnaires (based on Levels 1 and 2 of Kirkpatrick’s model) with qualitative post-intervention in-depth interviews, using a phenomenological approach and thematic analysis.

**Findings:**

The workshop yielded positive results, as evidenced by participants’ increased confidence to apply and use the information and skills acquired during the workshop. Critical success factors were as follows: participants were curiosity-driven; the use of multiple learning methodologies that attracted participants; and the use of authentic scenarios as a critical feature of the program.

**Originality/value:**

The intervention may offer a preliminary model for improving physician leadership skills in rural and remote settings by incorporating multiple teaching approaches and considering local cultural norms.

## Background

Primary care plays a leading role in providing health-care services in underserved communities. To continue to provide this service in the face of limited resources and infrastructure, however, physicians require strong leadership skills (Markuns *et al.*, 2010), especially in rural and remote settings. Hence, primary care physicians in these settings must be well-trained leaders who safeguard the continuous improvement and provision of comprehensive health care for their local community (Istiono *et al.*, 2015). Indeed, Richard *et al.* were already keen to point out that physicians in general, besides providing clinical care, must be able to collaborate with other health-care professionals and effectively lead a team in the patient-care setting and the broader context of health-care systems (Richard *et al.*, 2019). In low- and middle-income countries (LMICs), however, adequate education and training in leadership skills are often lacking (Tudor Car *et al.*, 2018). Consequently, most physician leaders rarely have any training or experience before being appointed to leadership or management positions. Sadly, the lack of such leadership capacity is increasingly cited as a significant constraint to the scaling up of priority interventions, especially in LMICs (Edmonstone, 2018).

When we look at the literature, we see a paucity of information about leadership development in underserved areas. Specifically, the current understanding of leadership skills relies solely on data from high-income countries in Europe, North America, and Australia, whereas little is known about how health-care providers apply these skills in LMICs (Scott *et al.*, 2016). Current findings in predominantly Western literature, show that physician leaders often feel unprepared for their leadership roles. Scholars, therefore, question whether traditional postgraduate training provides physicians with adequate education and training for such roles (Onyura *et al.*, 2019). As a result, it is suggested that formal training in the multifaceted components of leadership is needed and should begin at an early stage of a physician’s career. Unfortunately, to date, the number of comprehensive leadership training opportunities, at any career level, is limited (Sonnino, 2016).

In Canada, over 70% of health organisations adopted the LEADS Framework. The LEADS acronym stands for the following five leadership domains:

1. lead self;
2. engage others;
3. achieve results;
4. develop coalitions; and
5. systems transformation.

This framework provides a context for health-care leadership development by emulating leadership learning and practice. Furthermore, LEADS has been shown to be adaptable and practical in various organisations and settings (Dickson and Tholl, 2020). Therefore, we deemed it reasonable to assume that the framework also provides insight into physicians’ key capacities to carry out their duties, especially in rural and remote places in LMICs.

In the present study, we focused on Indonesia as an LMIC where studies on educational interventions to develop leadership skills among primary care doctors are scant, especially in the rural and remote context. Consequently, standard curricula or methods of instruction regarding leadership development are nonexistent. Using the example of Indonesia and, more specifically, the province of Aceh, we aimed to investigate how an educational intervention can impact the development of primary care doctors' leadership skills in a rural and remote context by identifying the pre-and post-intervention differences. This research focus is congruent with a recent Indonesian Ministry of Health decree presenting a primary health-care strategic roadmap that recognises the need for culturally sensitive and competent human resource performance to accomplish national health outcomes (Ministry of Health of the Republic of Indonesia, 2015). Therefore, understanding the impact of the aforementioned educational intervention in this specific setting would contribute to the successful and effective design of a program aimed at producing well-trained physician leaders in an LMIC context.

## Methods

### Study setting

The study was set in the province of Aceh, Indonesia, located at the northern end of Sumatera Island. While a fair share of Indonesian districts (29%) can be categorised as underdeveloped, nearly three-fourths of the country’s areas are rural (Noya *et al.*, 2021). Aceh province is one such area that is still largely underdeveloped. Divided into 18 districts and five autonomous cities (Wikipedia, 2021a), it is home to 2,244 villages that are still classified as developing and disadvantaged according to a ministerial decree (Ministry of Village Development of Disadvantaged Regions and Transmigration of the Republic of Indonesia, 2017). In 2020, the province recorded 359 primary care centres, 196 (55%) of which were in rural, 69 (19%) in remote and 33 (9%) in very remote areas (Aceh Health Office, 2020a).

We conducted the present study in North Aceh district, which has the highest density of sub-districts and villages in Aceh province, namely, 27 (Wikipedia, 2022) and 852 (Wikipedia, 2021b), respectively. Moreover, 254 of these villages are classified as underdeveloped (Ministry of Village Development of Disadvantaged Regions and Transmigration of the Republic of Indonesia, 2017). Furthermore, as the district with the most primary care centres, it is home to 32 such centres, 7 of which (21.9%) are located in remote areas, 24 (75%) in rural areas and 1 (3.1%) in an urban area (Ministry of Health of the Republic of Indonesia, 2021). Finally, it is also Aceh’s most densely populated district, with some 602,793 inhabitants in 2020 (Wikipedia, 2022). In contrast, it ranks among the ten Aceh districts/cities that in 2021 recorded the highest rate of poor population (Statistics Indonesia of the Province of Aceh, 2021).

### Study design

We developed an educational intervention consisting of a two-day workshop and evaluated its impact on participants’ knowledge and skills. A needs assessment of the province’s primary care physicians preceded the intervention's design. This was followed by further inquiry, based on which we determined the following:

- The intervention should revolve around five topics reflecting the most essential leadership traits as derived from the LEADS framework (see Appendix 1).
- The preferred leadership training format was a workshop facilitated by a lecturer from the faculty of medicine. Training sessions should ideally last for two days on average and should be evaluated using pre-and post-tests.
- As cultural difficulties present major and robust challenges to leadership in the rural health-care system, the intervention should be culturally sensitive (Maulina *et al.*, 2022). Indeed, Edmonstone already pointed out in his study that the development of leadership and management skills in non-Western societies must be acculturated; to ensure their user-friendly application, these skills must be conveyed and transferred across cultural boundaries (Edmonstone, 2018). We considered these recommendations in our design of the workshop.

### Workshop preparation

The main goal of our educational intervention program was to provide rural primary care physicians with a basic understanding of leadership knowledge and skills focused on cultural issues and local wisdom. Before the intervention, we conducted interviews with other stakeholders to refine the topics to be addressed in the workshop and to obtain a range of perspectives on the skills essential for working in rural or remote settings. The interviewees included:

- Two general practitioners who had worked in rural or remote primary care for at least 6 months in the North Aceh district and had a leadership role in their workplace (both were heads of primary care);
- Four trainees from the departments of pediatrics, gynecology, internal medicine and surgery (one person per department) who, before their speciality training, had worked in primary care or a hospital in one of the 18 districts of Aceh for at least six months;
- Three interns (doctors who practised medicine with guidance and supervision; before being allowed to practice independently, they were supervised for one year) who had been practising in rural or remote primary care in one of Aceh’s 18 districts; and
- Five medical students who had a rural or remote background in one of Aceh’s 18 districts.

We acquainted the interviewees with the LEADS framework, giving them a general overview and comparing the results from our previous study. By having this discussion, we obtained a more complete picture of what a well-rounded set of skills for a physician might look like and incorporated that into our educational intervention.

### Workshop content

The workshop covered five topics, each representing one of the five domains of the LEADS framework. The intended learning outcomes were based on our prior research findings (which, in turn, were guided by the LEADS framework), namely, the five most important leadership traits that rural/remote primary care doctors require. We also created a ‘workshop curriculum’, similar to an Indonesian-style “handy book”, containing the topics, main objectives, and intended learning outcomes. We shared this handy book three days before the workshop to familiarise participants with the LEADS framework and to allow them to view and study the module in advance.

### Workshop strategy

Throughout the workshop, we applied multiple learning techniques such as case-based discussion, debate and role-play, small-group discussion (group project), flipped classroom small-group discussion, and a sharing session. In doing so, we used an interactive teaching approach, applying the problem-based learning (PBL) method by offering different scenarios for each topic. To achieve our objectives, we recruited instructors who were experts, credible, well-experienced in leadership and had a background in medicine so that they could contribute insights from the perspective of physicians.

### Workshop evaluation

We evaluated the workshop in three ways using two different quantitative questionnaires, followed by qualitative interviews. The questionnaires were based on the LEADS framework (Dickson and Tholl, 2020) and publications on leadership training (Richard *et al.*, 2019). They were drafted in Indonesian and consisted of yes/no, multiple-choice, and a few open-ended questions. More specifically, the evaluation comprised:

- Post-intervention quantitative study to explore participants’ perceptions of the workshop’s quality. We developed the questionnaire according to Level 1 of Kirkpatrick's model, a widely known approach to training evaluation (Passmore and Velez, 2015). Participants completed the questionnaire at the end of workshop Day 2, indicating their level of agreement on a five-point Likert scale. The instrument was validated through face validity.
- Pre-and post-intervention quantitative study to measure participants’ self-reported learning. This time, we created the questionnaire following Level 2 of Kirkpatrick's model (Passmore and Velez, 2015). We administered the questionnaire before the workshop to obtain participants’ baseline data and at the end of Day 2 as quantifiable indicators of the learning that had taken place during the training. Participants were invited to rate their confidence level in about 15 specific leadership skills on a five-point Likert scale. The reliability of the instrument, as measured by Cronbach’s alpha, was 0.964.
- A post-intervention qualitative study to gauge participants’ workshop experiences and understand the workshop’s impact on physicians’ development as physician leaders was conducted seven days after the intervention. A phenomenological approach guided the interviews (see Appendix 2).

### Participants

**Figure 1.**
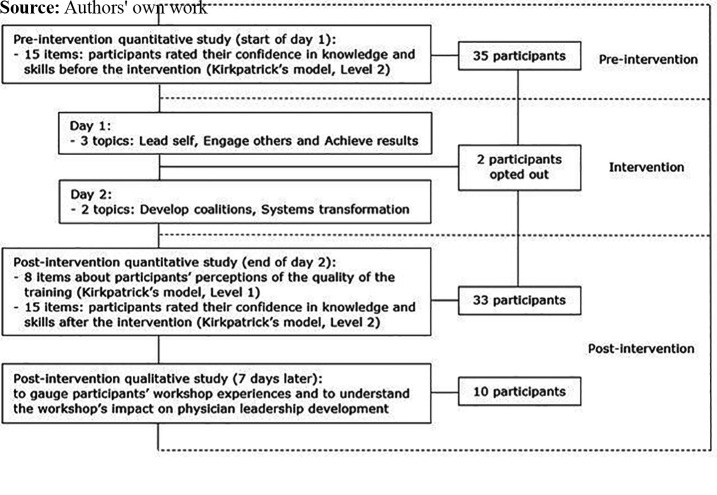
Flowchart of the workshop

The study sample comprised general practitioners in the North Aceh district who had worked in rural or remote primary care for at least six months. The said district counted a total of 87 doctors (Aceh Health Office, 2020b). We recruited 35 participants (see Figure 1) using the purposive sampling technique.

### Data analysis

To analyse our data, we first performed a descriptive analysis of the data about Level 1 of Kirkpatrick’s model using a graphic scale. We subsequently compared the pre-and post-test averages of each question from the Level-2 survey of Kirkpatrick’s model. Results were again visualised on a graphic scale. Finally, we performed a thematic analysis of the interview data.

### Ethical consideration

The research protocol was approved by the Medical and Health Research Ethics Committee of the Faculty of Medicine, Public Health and Nursing at Universitas Gadjah Mada/Dr Sardjito General Hospital, Yogyakarta, Indonesia, Ref. No. KE/FK/0609/EC/2021.

### Reflexivity

JB is a pediatrician, educationalist and associate professor of medical education with specific expertise in equity and leadership development in postgraduate medicine. FS is a practising gynecologist and a professor of health systems innovation and education in Amsterdam; he specialises in qualitative approaches in cross-disciplinary research projects. MH is a medical doctor and an associate professor of public health in Indonesia, where he teaches primary health-care policy and management. Finally, FM is an Aceh-based general practitioner whose research interests include physician leadership in underprivileged communities and health-care systems in rural and remote settings.

The authors were cognisant of the potential for bias in qualitative investigations, especially because the lead researcher (FM) is from the environment under investigation, and adjusted their research approach accordingly. However, despite this potential risk, the authors felt that the diverse composition of their study team would suitably mitigate any such bias. With their experience as physician leaders and researchers in The Netherlands, FS and JB, for instance, were able to offer a complementary, outside (Western) European perspective on the study design, data analysis, and interpretation. In doing so, they were able to debunk any potentially emerging myths. Additionally, as a senior researcher from Indonesia, MH oversaw the process by providing crucial suggestions based on his knowledge of local and cultural dynamics.

## Findings

### Demographic characteristics

The participants were 33 primary care physicians (Table 1). Regarding prior leadership training, most participants indicated that they had not received any leadership training during their undergraduate education or in their current workplace. Yet, over half of them held leadership positions in their current workplace, such as head of primary care, quality assurance, individual services, or community services.

### Post-intervention quantitative study

**Figure 2.**
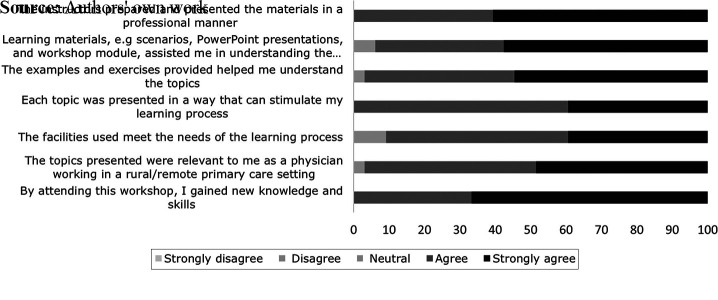
Participants’ perceptions of the workshop quality, as measured by Kirkpatrick’s satisfaction survey (expressed as percentages)

The 33 participants completed the survey (Kirkpatrick, Level 1). Their ratings of the instructors, learning materials, examples and exercises, presentation of topics and facilities were overwhelmingly positive (agree/strongly agree). Similarly, participants indicated that the topics were relevant to them as physicians working in rural or remote settings and that they had gained new knowledge and skills by attending the workshop. A few of them also gave “neutral” ratings to some categories (learning materials, examples and exercises, facilities and relevance of topics). At the same time, nobody disagreed or strongly disagreed with any of the statements (see Figure 2).

Concerning participants’ overall evaluation of the workshop, we found that 75.8% (25/33) rated the workshop as “very good”, and 24.2% (8/33) rated it as “good”. In contrast, nobody gave it a “bad” or “very bad” evaluation (Table 2).

### Pre- and post-intervention quantitative study

**Figure 3.**
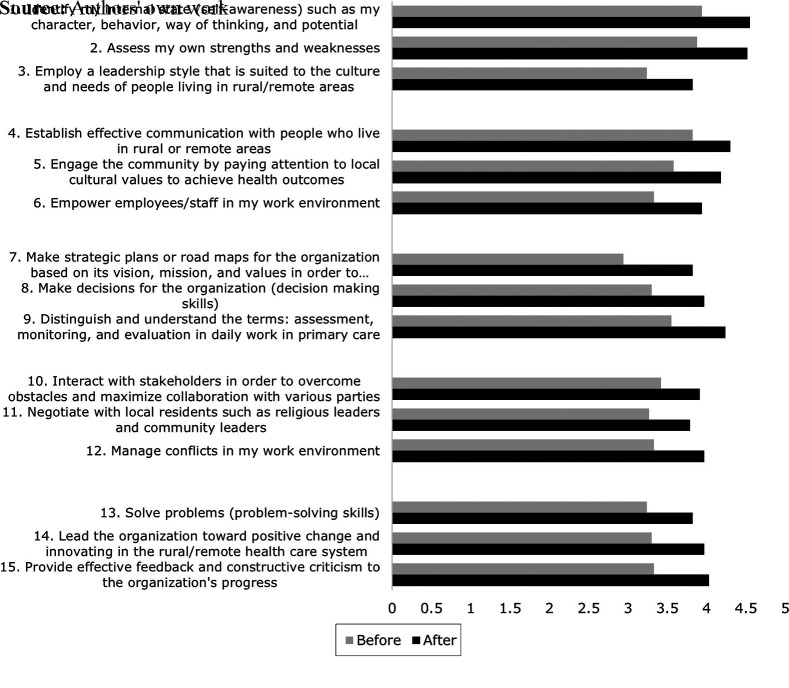
Participants’ evaluation of the workshop’s influence on their knowledge and skills as measured by their confidence in the statements listed, ranging from not at all confident to very confident (presented as means per item). Statements 1-3 represent the Lead self domain; statements 4-6 represent the Engage others domain; statements 7-9 reflect the Achieve results domain; statements 10-12 concern the Develop coalitions domain; and statements 13-15 appertain to the Systems transformation domain

Our comparison of the pre-and post-intervention questionnaire results about participants’ learning processes (Kirkpatrick, Level 2) revealed that participants gained more confidence to apply and use the knowledge and skills related to leadership capacity. As shown in Figure 3, the statement that presented the highest average increase (of 0.88 points) was to “make strategic plans or road maps for the organization based on its vision, mission and values to achieve results using a local cultural approach” (Achieve results domain). The score on this statement rose from 2.94 before the workshop to 3.82 after the workshop. The second-highest increase was observed in the Systems transformation domain, with an average 0.7 point increment for the ability to “provide effective feedback and constructive criticism about the organization’s progress”. Finally, the Achieve results domain again provided the third-highest increment (of 0.69 points), specifically in participants’ confidence to “distinguish and understand the terms: assessment, monitoring, and evaluation in daily work in primary care”.

Conversely, we identified three statements with the smallest improvement in participants’ learning. The first concerned participants’ ability to “establish effective communication with people who live in rural or remote areas” in the Engage others domain, which rose by a mere 0.48 point from 3.82 before to 4.3 after the workshop. The second and third smallest improvements amounted to 0.49 point and 0.52 point, respectively, and applied to the ability to “interact with stakeholders to overcome obstacles and maximise collaboration with various parties” and to “negotiate with residents such as religious and community leaders”, both from the “Develop coalitions” domain. Hence, we concluded that workshop participation activated an effective learning process by enhancing participants' confidence to apply and use the knowledge and skills related to leadership capacity.

### Post-intervention qualitative study

#### Demographic findings and motivation to participate.

We interviewed ten primary care physicians (Table 3). Concerning the previous instruction, most respondents had not received any leadership training in their undergraduate education or their current workplace. Yet, seven did hold leadership positions in their current workplace, such as head of primary care, chief of individual services in primary care, and chief of quality assurance. Participants were intrinsically motivated to participate in the workshop, driven by their curiosity about leadership.

Based on the interviews, we identified two main themes: workshop feedback and evaluation of learning outcomes, and several subthemes that reflect participants’ workshop experiences.

### Workshop feedback

#### Dynamic workshop format.

All respondents stated that they enjoyed the workshop because of the variety of learning methods used, which made it less monotonous:

This workshop was not monotonous [because in this kind of training participants generally only read PowerPoint slides]. The instructors showed examples and gave short explanations. This workshop was very good because there was always room for interaction or discussion. There were also sessions with feedback from participants and instructors, and the atmosphere was lively and did not make us sleepy. [Participant 1]

The method provided helped participants understand the topics very well. After the group discussion, we gave a presentation of what we learned. Participants could say what they think. It helped us see things from different points of view, so we didn't only consider our perspective. I enjoyed the discussion very much. [Participant 9]

Moreover, participants expressed their appreciation of the scenarios, for they resembled their daily work activities in real life. We encouraged participants to solve the problem cases, hoping that they could be a trigger for learning. Participants acknowledged that:

The dilemmas in the scenarios were comparable to how we feel when working in rural or remote primary care. [Participant 3]

By discussing the problems in scenarios, I practised my knowledge and skills. [Participant 7]

Not only did participants discuss the scenarios, but they also shared their experiences. Some participants who had a leadership role, such as head of primary care, were happy to share their leadership experiences in their workplace:

I learned from several discussions with multiple participants who had been heads of rural or remote primary care for many years. Their experiences provided various inputs and solutions to the scenarios. I learned from them as well. [Participant 2]

#### Inspiring.

Other participants mentioned that the workshop had inspired them to create a program in their primary care practice aimed to promote behaviour change and improve patient outcomes:

I was inspired to ask tuberculosis patients who have been cured to become cadres in our primary care and act as role models for other tuberculosis patients so they would be eager to take medicine for 6–9 months and recover. [Participant 2]

[…] inspired me to improve teamwork and cross-sector collaboration to implement a healthy latrine program [some people in remote villages still defecate in the river for cultural reasons]. [Participant 5]

I intend to invite religious leaders and hold a discussion [about stunting] with them at the mosque after Friday prayers [the day on which all stakeholders gather in the mosque]. I will also invite the village head, “Tuhapeut” [who has a respected position in Aceh's rural communities], and the subdistrict head. [Participant 6]

### Evaluation of learning outcomes

#### Mindset shift.

In addition to the workshop being a source of inspiration, participants also reported that it had brought about a shift in their mindset. While unfamiliar at first with the LEADS framework through which we introduced the leadership concept, participants were intrigued by it – so much so that they changed their thinking about leadership:

Everything starts with the self [lead self]. It could even be that each of us already has the soul of a leader but doesn’t know it. Even though we don't have a position in primary care management, we can involve other people [Engage others domain] and build coalitions [Develop coalitions domain], which is part of what it means to be a leader. [Participant 4]

[…] changed my mind about how we as doctors must be able to lead. [Participant 9]

Someone with a leadership role is not always a leader and vice versa. [Participant 10]

Furthermore, workshop attendance made respondents consider the pursuit of a leadership role, for they believed that they would be able to accomplish more:

I began to believe that if we were leaders in primary care rather than just doctors in the service unit, we could accomplish much more. Because only the leader has the authority to implement particular policies or make decisions. So, if you only focus on being a doctor in the service unit, you will not be able to accomplish much. [Participant 1]

I wanted to be the head of primary care, but not out of personal ambition. I want to contribute and change [primary care] for the better. Integrity was something not everyone had. [Participant 4]

Finally, respondents also felt they had gained new perspectives/knowledge about physician leadership. It was not easy for them to be a primary care doctor in a rural/remote setting; most of the things they needed to know, they simply learned by doing. The workshop, therefore, offered a welcome stepping stone:

I began to be aware of my abilities, how capable I was to lead [people], and that was the basis for me to be able to move [work]. I plan to communicate and collaborate with them [stakeholders]. [Participant 8]

#### Dare to opine.

Most respondents became more willing to voice their opinions, especially by giving suggestions on improving access to health services for patients. The following respondent, for instance, became more daring to speak up to her leader:

[…] how to convince them [primary care management] so patients can get better care; I was more confident. It was not for my benefit, but for the patients’. I was more willing to express my viewpoint to the primary care leader. [Participant 1]

Another respondent also said that she felt better able to communicate with staff and patients in primary care:

[…] better at communicating and beginning to offer solutions. I also started inviting patients to more extensive discussions. [Participant 9]

Importantly, workshop participation made some respondents more aware of and concerned about their workplace environment, patients and the community:

I was a selfish person who did not care about what happened around me. Now, I was motivated to observe my work environment and patients’ needs. [Participant 2]

#### Action-confident.

We identified that the majority of participants (9/10) became more confident. As previously stated, only a few participants had received prior leadership training. The impact of the workshop was such that after participation they felt more confident about their ability to lead people:

Before […] I often had doubts, felt uneasy, and worried. Now, I feel more confident, especially in providing health services to the communities. [Participant 1]

I felt more sure of myself and my ability to run the program. [Participant 3]

We also discovered that participants felt more confident in making decisions and they were more often inclined to take the initiative:

I was more willing to make decisions. I was more open-minded about decisions. [Participant 10]

I directed and asked village cadres to be more actively involved in our primary care program. I also provided them with some basic training. [Participant 3]

## Discussion

### Why did the workshop attract participants?

Our study examined how an educational intervention affected primary care doctors' leadership skills in Aceh, Indonesia. We observed that the intervention succeeded for the following three reasons:

1. Driven by their curiosity, participants were intrinsically motivated to participate. As leadership training was limited in the setting under scrutiny, the majority of participants had only received training that was clinical in nature, thereby rendering the workshop a fresh and inviting experience.
2. We used multiple learning approaches across sessions, which helped retain participants’ attention.
3. The program revolved around the use of authentic scenarios.

Our workshop was an effective way to build leadership competencies because of the complex interplay between internal (e.g. participants’ extensive work experience and adjustment to systems) and external factors (e.g. the learning approaches used).

### Curiosity as intrinsic motivation

Leadership development is scant, especially in developing countries in rural and remote areas. This certainly holds for Indonesia, where physicians generally only receive such training from the local health office and mostly when appointed to a leadership position, such as head of primary care. Most of our study participants had never attended a program specially designed for rural and remote primary care doctors that essentially focused on developing leadership skills. They were, therefore, very keen to participate in our workshop, which aroused their curiosity. Having construed this curiosity as one of the workshop’s success factors, we decided to study leadership through curiosity. “Curiosity” has been defined in the literature as a drive to learn new things and to have new sensory experiences, which encourages exploratory action. We observed that our participants enjoyed asking and discussing questions during the workshop, which has been identified as an essential marker of curiosity (Guo *et al.*, 2010). Curiosity is associated with a passion for learning (Borowske, 2005). Yet another study suggested that women can display curious behaviour by actively studying the past, challenging the present, and conferring with their trainer or mentor about their respective experiences (Wagstaff *et al.*, 2021). This is precisely what our study participants – predominantly women – did: they were more likely to explore and compare their current situation to past experiences. From another study, we also learned that curiosity might be considered a crucial trait and competency that equips leaders with the right mindset to grasp the organisation’s needs and make the best decisions (Yen, 2013). Finally, prior research has defined curiosity as a catalyst for leadership, as a professional and organisational norm and as a catalyst for organizing (Lievens *et al.*, 2022).

### Multifarious approaches to learning

The second factor in the workshop's success was our use of multiple learning methods, which included case-based discussion, debate and role-play, a group project, small-group discussion and presentations (in the form of mind mapping) and a sharing session. In our experience, these approaches were appropriate for adult learners, including primary care physicians. What also helped render the workshop successful was the participants’ length of employment. While most had worked in rural or remote primary care for 2–10 years, one-third had over 11 years of experience. As such, they were not “newcomers” to the rural or remote health-care system and knew what practising in these environments was like. Indeed, previous research has demonstrated that adults can draw from their previous learning experiences to create learning activities, and serve as resources for one another during learning events. In this process, they frequently need to modify, transfer, and re-integrate meanings, values, strategies, and skills (Jackson and Macisaac, 1992). By using a multitude of learning methods with discussion/interaction, we at least expected our participants to:

- develop critical thinking by integrating their past experiences and own thoughts to generate a well-informed conclusion;
- enhance verbal communication skills by expressing their opinions and ideas during the workshop; and
- explore their ideas and creativity, for instance, by drawing their ideas on a mind map (in Topic 4).

This so-called mind mapping is a learning technique by which participants examine and explore different concepts by linking peripheral branches to a central topic using various relationships (Rezapour-Nasrabad, 2019). Our findings are consistent with those from a study by Massachusetts Medical School on leadership training for medical students. Equally based on the student-centred learning paradigm in which student-led discussions were vital, the study reported higher levels of student engagement, personalised learning and personal application (Richard *et al.*, 2019).

### Real-life-based scenarios

A final ingredient that proved instrumental in making the workshop succeed was our use of PBL based on authentic scenarios. Intended to be a trigger for learning, the scenarios were derived from real stories. More specifically, in designing the problem cases, we kept in mind that they should:

- be relevant (tailored to participants’ level of knowledge and experiences);
- resemble real-life situations;
- apply to future practice; and
- attract learners’ attention more than lecture-based approaches do.

Moreover, the scenarios were designed to promote:

- the development of problem-solving skills and independent and active learning;
- decision-making about the way to explore the problem, which enhances critical thinking and challenges participants’ prior knowledge; and
- teamwork building by discussing and examining the scenarios together with other participants.

We observed that our scenarios were an effective means for participants who had worked in constrained environments for many years: they were already familiar with the patterns of behaviour existing in rural or remote primary care practice and its communities, such as their rules, procedures, prevailing habits and structures (Gajda, 2019). Moreover, they had good environmental adaptability because they had already grown accustomed to working with limited resources, rural or remote populations, and local cultures and adapted accordingly.

Lastly, our study also revealed that, despite their unfamiliarity with the concept, our participants had already implemented many of the leadership traits outlined in the LEADS framework. What they learned in the workshop closely resembled what their rural/remote practice situation required, such as interacting with the local communities and negotiating with their leaders. Bearing in mind the above-listed design criteria, we, therefore, expected our participants to be able to apply the leadership skills gained during the workshop to their work in constrained environments. Their previous work experience allowed participants to recognise and accept their environment, which, in our view, was crucial to empowering them to help improve the rural/remote health system and associated health outcomes.

As with any study, our study has some limitations. First, we based the evaluation on self-assessments rather than objective assessments. A combination of subjective and objective measurements would have been desirable to appreciate the workshop's success more comprehensively, especially in resource-limited settings. Second, our study sample was limited and only represented one of the 18 districts in Aceh province.

## Conclusions

Our research on physician leadership skills development in Aceh province, Indonesia, offers a preliminary model for developing a physician leadership program in rural and remote settings that uses multiple teaching approaches and considers local cultural values. Based on our positive findings, we conclude that our intervention can serve as an example or source of inspiration to faculty developers wanting to improve physicians’ leadership skills, particularly among future rural and remote primary care doctors who work in an LMIC context.

## Figures and Tables

**Table 1.** Participant characteristics (*N* = 33)

Characteristic	Total (*n*)	%
*Age (in years)*		
25–35	17	51.5
36–45	15	45.5
46–55	1	3.0
*Gender*		
Male	3	9.1
Female	30	90.9
*Type of primary care*		
Rural	26	78.8
Remote	7	21.2
*From a rural/remote background*		
No	4	12.1
Yes	29	87.9
*Type of university*		
Public	16	48.5
Private	17	51.5
*Experience in medical practice (in years)*		
1–5	13	39.4
6–10	8	24.2
11–15	9	27.3
16–20	2	6.1
>20	1	3.0
*Work experience in rural/remote practice (in years)*		
1–5	16	48.5
6–10	9	27.3
11–15	6	18.2
16–20	1	3.0
>20	1	3.0
*Received leadership training in undergraduate education*		
No	31	93.9
Yes	2	6.1
*Received leadership training in the current workplace*		
No	25	75.8
Yes	8	24.2
*In a leadership role in my current workplace*		
No	10	30.3
Yes	23	69.7

**Source:** Authors’ own work

**Table 2.** Participants’ overall workshop ratings (Kirkpatrick, Level 1)

Overall evaluation	Very bad (%)	Bad (%)	Neutral (%)	Good (%)	Very good (%)
I rate this workshop as	0	0	0	8 (24.2)	25 (75.8)

**Source:** Authors own work

**Table 3.** Participant characteristics (*N* = 10)

Characteristic	Total (*n*)
*Age (in years)*	
25–35	5
36–45	4
46–55	1
*Gender*	
Male	2
Female	8
*From a rural/remote background*	
No	2
Yes	8
*Type of university*	
Public	6
Private	4
*Experience in medical practice (in years)*	
1–5	3
6–10	2
11–15	3
16–20	1
>20	1
*Work experience in rural/remote practice (in years)*	
1–5	4
6–10	3
11–15	1
16–20	1
>20	1
*Received leadership training in undergraduate education*	
No	9
Yes	1
*Received leadership training in the current workplace*	
No	7
Yes	3
*Holds a leadership position in the current workplace*	
No	3
Yes	7

**Source:** Authors’ own work

**Table A1.** Workshop outline

Day	Domain	Subdomain	Topic	Main objectives – Participants will have the ability to…	Intended learning outcomes – Participants…	Outline
1	Lead self	1. Manage themselves 2. Demonstrate character 3. Develop themselves 4. Be genuine and passionate 5. Be self-aware	Being a leader	1. identify internal self-awareness and its importance in leadership 2. assess their strengths and weaknesses 3. identify individual leadership styles (appropriate to the culture and local needs)	1. can take responsibility for their performance and health 2. model qualities such as honesty, integrity, resilience and confidence 3. actively seek opportunities and challenges for personal learning, character-building, and growth 4. are genuine and passionate 5. are aware of their strengths and limitations	• 10:00–10:15: introductiona,^c^ • 10:15–11:45: case-based discussion (Scenario 1) • 11:45–12:00: wrap-upb • 12:00–13:00: break
1	Engage others	1. Foster the development of others 2. Communicate effectively 3. Build teams 4. Contribute to the creation of healthy organisations 5. Support community-driven processes	Communicating effectively	1. establish effective communication with rural or remote communities 2. apply cultural/local contexts to achieve health outcomes 3. empower employees/staff	1. support and challenge others to achieve professional goals 2. listen well and encourage an open exchange of information and ideas using appropriate communication media 3. facilitate environments of collaboration and cooperation to achieve results 4. create engaging environments in which others have meaningful opportunities to contribute 5. want to understand the commitment being made and be assured it will have a positive effect on their community	• 13:00–13:15: introductiona • 13:15–14.45: Debate and role-play (Scenario 2) and presentation • 14.45–15:00: wrap-upb and break
1	Achieve results	1. Take action to implement decisions 2. Set the direction 3. Assess and evaluate 4. Strategically align decisions with vision, values and evidence 5. Promote community-centered care	Attaining goals	1. make vision-, mission- and value-inspired roadmaps for performance-oriented results based on a cultural approach 2. make informed decisions 3. understand and differ: assessing, monitoring and evaluating health outcomes	1. act in a manner consistent with organisational values to provide effective and efficient public-centered service 2. inspire vision by identifying, establishing and communicating clear and meaningful expectations and outcomes 3. assess and evaluate outcomes 4. integrate organisational missions and values with reliable, valid evidence to make decisions 5. understand that cultural safety is more than a history lesson (it is about opening dialogue with many different people about wellness; in doing so, the leader creates the appropriate conditions for this dialogue and joint learning)	• 15:00–15:15: introductiona • 15:15–16:45: flipped group discussion/group project (2015–2022 strategic plan document of Aceh/Aceh Health Office road map) and presentation • 16.45–17:00: wrap-upb
2	Develop coalitions	1. Purposefully build partnerships and networks to create results 2. Demonstrate a commitment to customers and service 3. Navigate socio-political environments 4. Mobilise knowledge	Building powerful coalitions	1. proactively engage with stakeholders to overcome barriers and maximise collaboration 2. understand the power of negotiation (particularly with local inhabitants such as religious and local leaders) 3. manage conflicts	1. create connections, trust and shared meanings with individuals and groups 2. facilitate collaboration, cooperation and coalitions among diverse groups and perspectives to improve service 3. show a political astuteness 4. encourage an open exchange of information 5. employ methods to gather intelligence	• 10:00–10:15: introductiona • 10:15–11:15: small-group discussion (mind mapping) and presentation • 11.15–11.30: wrap-upb and break
2	Systems transformation	1. Demonstrate systems/critical thinking 2. Orient themselves strategically to the future 3. Encourage and support innovation	Leadership and systems transformation	1. broaden their perspective on issue-framing and problem-solving approaches 2. bring about positive change and innovate rural health systems 3. give effective feedback and constructive criticism	1. can identify issues, solve problems and design and implement effective processes across systems and stakeholders 2. question and challenge the status quo (thereby effecting positive change and fostering innovation) 3. scan the environment for ideas, best practices and emerging trends (and, in so doing, make maximum use of the resources available) 4. create a climate of continuous improvement and creativity aimed at systemic change 5. think analytically and conceptually	• 11:30–11.45: introductiona • 11.45–12.45:small-group discussion (of Scenario 3) • 12.45–13.45: a GP from Eastern Indonesia shares experiences, followed by a discussion • 13.45–14.00: wrap-upc

**Notes:** ^a^Participants are welcomed and presented with an overview of the topic, main objectives and intended learning outcomes; ^b^consists of closing remarks; ^c^participants receive instructions to complete a pre-test or post-session questionnaire

**Source:** Authors’ own work

## Appendix 1

Table A1

## Appendix 2

Interview guide based on Kirkpatrick’s model, Level 2 (post-intervention qualitative study).

### Interview guide

1. Do you have any feedback regarding methods, learning materials (scenarios, PowerPoint presentations and workshop manual), examples and exercises?
2. Do you put what you learned in training to use in your daily work? To what extent were the knowledge and skills gained during the training program applied in the workplace?
3. How are you performing in your current role compared with how you performed after past training sessions?
4. What personal or professional gains and/or rewards have you experienced in your work as a result of applying the knowledge gained from the training?

**Source:** Authors’ own work
